# Non-canonical transcriptional regulation of heme oxygenase in *Aedes aegypti*

**DOI:** 10.1038/s41598-019-49396-3

**Published:** 2019-09-24

**Authors:** Vanessa Bottino-Rojas, Luiza O. R. Pereira, Gabriela Silva, Octavio A. C. Talyuli, Boris C. Dunkov, Pedro L. Oliveira, Gabriela O. Paiva-Silva

**Affiliations:** 1 Instituto de Bioquímica Médica Leopoldo de Meis, Universidade Federal do Rio de Janeiro, Rio de Janeiro, 21941-902 Brazil; 2 Laboratório de Pesquisas em Leishmaniose, Instituto Oswaldo Cruz, FIOCRUZ, Rio de Janeiro, 21040-360 Brazil; 3 Center for Insect Science, The University of Arizona, Tucson, AZ 85721-0106 USA

**Keywords:** Transcription, Metabolism

## Abstract

Heme oxygenase (HO) is a ubiquitous enzyme responsible for heme breakdown, which yields carbon monoxide (CO), biliverdin (BV) and ferrous ion. Here we show that the *Aedes aegypti* heme oxygenase gene (AeHO – AAEL008136) is expressed in different developmental stages and tissues. AeHO expression increases after a blood meal in the midgut, and its maximal transcription levels overlaps with the maximal rate of the further modified *A*. *aegypti* biglutaminyl-biliverdin (AeBV) pigment production. HO is a classical component of stress response in eukaryotic cells, being activated under oxidative stress or increased heme levels. Indeed, the final product of HO activity in the mosquito midgut, AeBV, exerts a protective antioxidant activity. AeHO, however, does not seem to be under a classical redox-sensitive transcriptional regulation, being unresponsive to heme itself, and even down regulated when insects face a pro-oxidant insult. In contrast, AeHO gene expression responds to nutrient sensing mechanisms, through the target of rapamycin (TOR) pathway. This unusual transcriptional control of AeHO, together with the antioxidant properties of AeBV, suggests that heme degradation by HO, in addition to its important role in protection of *Aedes aegypti* against heme exposure, also acts as a digestive feature, being an essential adaptation to blood feeding.

## Introduction

*Aedes aegypti* is a vector of important human viral diseases such as yellow fever and dengue, and more recently has also been associated with the large-scale emergence of viruses such as Chikungunya^1^ and Zika^2^. Both adult males and females feed on nectar, but only females require the ingestion of large amounts of vertebrate blood, as a nutritional source for oogenesis. Blood is a protein-rich diet that elicits rapid signaling responses that include nutritional and endocrine regulation^3^. Since hemoglobin is the main blood protein, like other hematophagous animals, mosquitoes face a unique circumstance regarding its digestion, resulting in the formation of large amounts of heme^4^. Besides its relevance in many physiological processes, free heme is able to amplify formation of reactive oxygen species (ROS)^5^ and to destabilize the membrane structure leading to cell lysis^6^. Due to the potential toxicity of free heme, the intracellular levels of this molecule are strictly controlled by the balance of the specific biosynthesis and degradation pathways^7^.

In most eukaryotic cells, heme oxygenase (HO) catalyzes the degradation of heme, cleaving its alpha-meso carbon bridge to yield equimolar quantities of biliverdin IX alpha, CO and free iron^7^. Free iron is promptly sequestered into ferritin and, in mammals, biliverdin is subsequently converted to bilirubin through the action of biliverdin reductase^8^. Thus, heme oxygenase is a major component of cellular response against stress.

Very little is known about the cellular mechanisms involved in heme degradation in blood-feeding insects. The heme degradation pathway has only been described in the mosquito *A*. *aegypti*^9^ and in the hemipteran *Rhodnius prolixus*^10^, vector of Chagas disease. In both cases heme degradation process displays several peculiarities when compared to vertebrates pathway. In general, these distinctive features relate to the regular ingestion of heme associated with reproduction. Further modifications of biliverdin, with the conjugation of hydrophilic molecules, facilitates the completion of heme degradation and the removal of massive amounts of otherwise hydrophobic biliverdin molecules^4^.

In *R*. *prolixus*, we have previously shown the existence of a unique pathway for oxidative heme degradation, which produces a modified dicysteinyl-biliverdin IX gamma as the main end-product^10^, and not the BV IX alpha as in the vertebrates. In the mosquito *A*. *aegypti*, heme enzymatic degradation has also been shown to occur and, differently from all other previously studied organisms, the produced biliverdin is further modified by conjugation of two glutamine residues, leading to the formation of an excretable biglutaminyl-biliverdin IX alpha (or *Aedes aegypti* biliverdin, AeBV)^9^.

Thus far, three isoforms of HO, HO-1, HO-2 and HO-3 have been described. HO-2 isoform is constitutively expressed, HO-1 is induced by multiple stress stimuli whereas HO-3, only described in *Rattus novergicus*, is considered to be catalytically inactive^11^. In insects, HO have only been functionally analyzed in the fruit fly *Drosophila melanogaster*^12^ and the hematophagous mosquito *Anopheles gambiae*^13^. However, the physiological relevance of HO and of its product BV IX as antioxidant components and the regulation of HO expression in the context of adaptation to hematophagy have not been investigated so far.

Taking into account that heme degradation is a priority for cells that are exposed to its high levels, the present study sets to fill the gap in the characterization of this relevant pathway pathway in the blood feeding mosquito *A*. *aegypti*. Here we examined, for the first time in a hematophagous insect, the expression of the Heme oxygenase, the key enzyme responsible to the heme degradation. The *A*. *aegypti* HO (AAEL008136) gene encodes a predicted protein with high degree of similarity with classical heme oxygenase sequences. The transcriptional profile was evaluated in parallel with the formation of AeBV, its antioxidant product. Against all odds, our results show that the *A*. *aegypti* HO gene is transcriptionally regulated by a nutrient-sensing pathway and not by stress or heme imbalance, as described for other organisms up to now.

## Results

### The *Aedes aegypti* heme oxygenase (AeHO)

**Figure 1:**
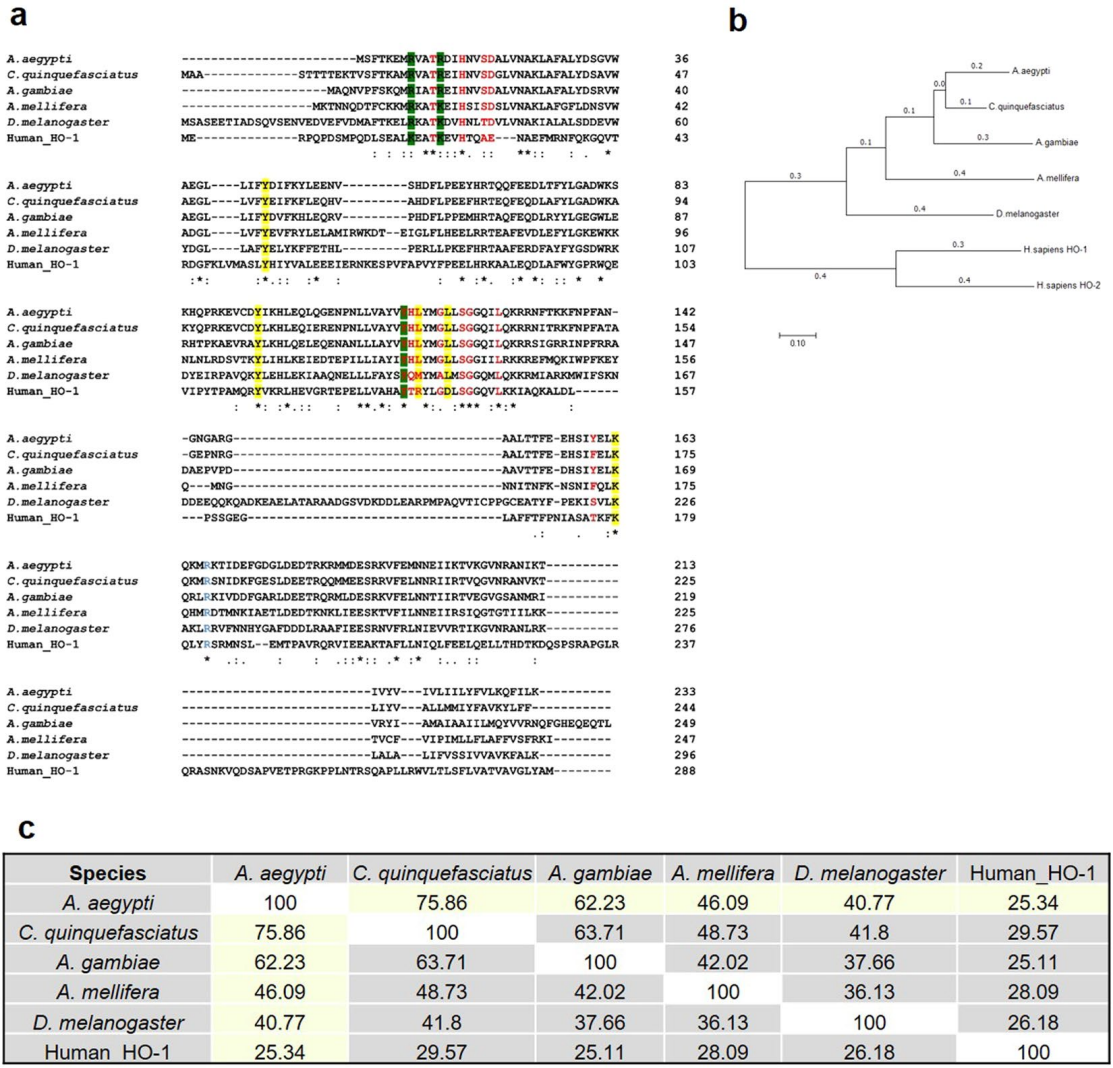
Heme oxygenase amino acid sequence. (**a**) Alignment of HOs from several insects and human HO-1, comparing the most important residues in the heme interaction. Residues that contact to heme (red); hydrophobic residues wall (blue); residues that exhibit interaction with propionates (green box) and polar residues clusters (yellow box). (**b**) Neighbor-Joining phylogenetic tree for insects and human HOs. The optimal tree with the sum of branch length = 3.03131229 is shown. The tree is drawn to scale, with branch lengths (next to the branches) in the same units as those of the evolutionary distances used to infer the phylogenetic tree. The evolutionary distances were computed using the Poisson correction method and are in the units of the number of amino acid substitutions per site. (**c**) Percent identity matrix based on alignment (Clustal Omega).

The gene sequence for heme oxygenase from *A*. *aegypti* (GenBank accession number AAEL008136) has a 988 bp ORF, which encodes a predicted 233 amino acid polypeptide (Fig. 1a). Only one HO-1 predicted ortholog was found in the *A*. *aegypti* genome (VectorBase Genome Assembly: AaegL5), suggesting that this mosquito expresses only one isoform of HO. No evidences of more than one transcript were found in the microarrays and RNA-Seq experiments results deposited in the VectorBase database (www.vectorbase.org). The deduced amino acid sequence of AeHO showed high similarity to sequences of heme oxygenases from other insects and also to the inducible human isoenzyme HO-1 (Fig. 1b). The percentage of identities/similarities between AeHO and HO amino acid sequences in other insects and humans are shown in Fig. 1c. From the alignment we were able to identify conserved residues relevant for enzymatic activity and for building of the heme pocket of the catalytic site^14^, such as the proximal heme ligand His25. Most of the residues involved in the interaction with the heme propionate residues localized in the amino terminal portion are also conserved. In HO-1, a hydroxyl group is the distal heme ligand and establishes hydrogen bonding to Gly139 and Gly143, which are conserved in insect HOs, with the exception of *Drosophila melanogaster* HO. The fly HO lacks a proximal heme ligand resulting in the production of a broad spectrum of BV isomers (α, β, δ)^12^.

### The AeHO expression and heme degradation

**Figure 2:**
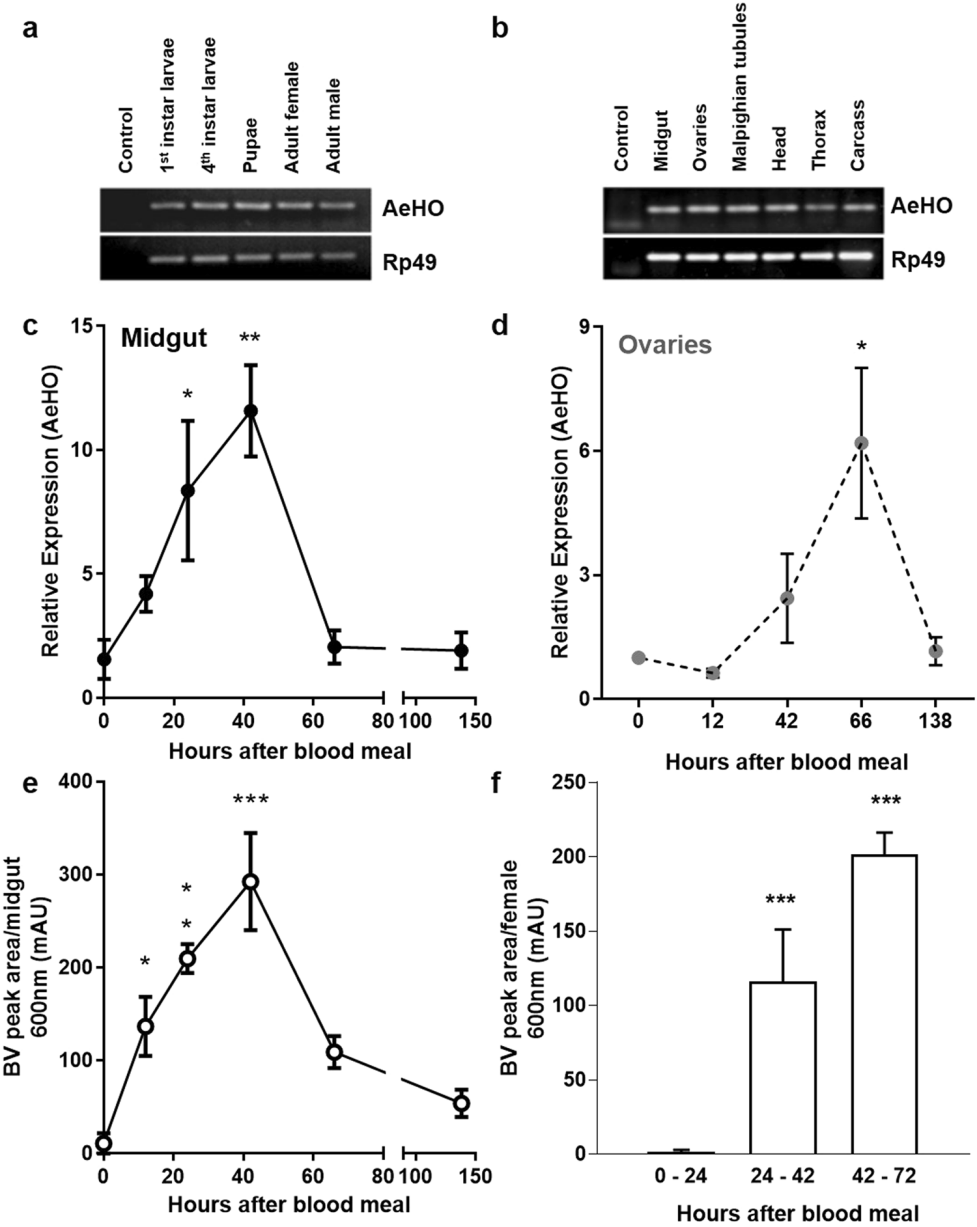
*Aedes aegypti* heme oxygenase - expression profile and production of biliverdin. (**a**) Reverse transcription PCR (RT-PCR) revealing heme oxygenase expression from early developmental stages to adults. (**b**) RT-PCR showing HO expression in female tissues dissected 24 h after the blood meal. Gel images were cropped and processed to improve clarity – original full-length gels are presented in Supplementary Fig. S2. (**c**) Quantitative PCR (Real time qPCR) showing the time course of HO expression in the midgut and (**d**) in the ovary. Rp49 was used as endogenous control (n = 4). (**e**) Time course of AeBV production in the midgut. (**f**) Appearance of AeBV in mosquito feces. Results of (**e**) and (**f**) are pools of at least 3 independent experiments – mAU, 1000x arbitrary units. Error bars indicate the standard error of the mean; one-way ANOVA with Dunnett’s post-test with multiple comparisons for (**c**), (**d**), (**e**) and (**f**), where 0 h or 0–24 h after the blood meal samples served as control.

In order to address the importance of this gene in the mosquito *A*. *aegypti*, we initially investigated its broad transcriptional profile, that included the non-hematopagous stages (larvae, pupae and adult males) of mosquito, as HO is expressed in many tissues in vertebrates, including the ones that are not intensively involved in heme detoxification. AeHO is expressed at different developmental stages as well as in many tissues of blood fed females (Fig. 2a,b). As the trigger of oogenesis event is strictly dependent of blood ingestion and digestion in this insect, we compared the expression of AeHO in midguts and ovaries of blood-fed females. The AeHO transcript levels increased in midgut and ovaries after a blood meal (Fig. 2c,d). In the midgut, AeHO transcripts increased between 24 h and 42 h after the blood meal (Fig. 2c), a period of maximum heme concentration in the midgut lumen^15^, whereas the maximal expression of AeHO in the ovary occurred between 48 h and 72 h, when the oogenesis is getting into the end in females mosquitoes (Fig. 2d). The amount of AeBV, the end product of the heme degradation pathway, increased in the gut until 24 h after the blood meal, then declined (Fig. 2e) as a result of its appearance in the insect feces (Fig. 2f). Due to the relevance of biliverdin production as a result of HO activity after blood ingestion, we confined the further analyses to the midgut of blood-fed females.

### Antioxidant activity of AeBV

**Figure 3:**
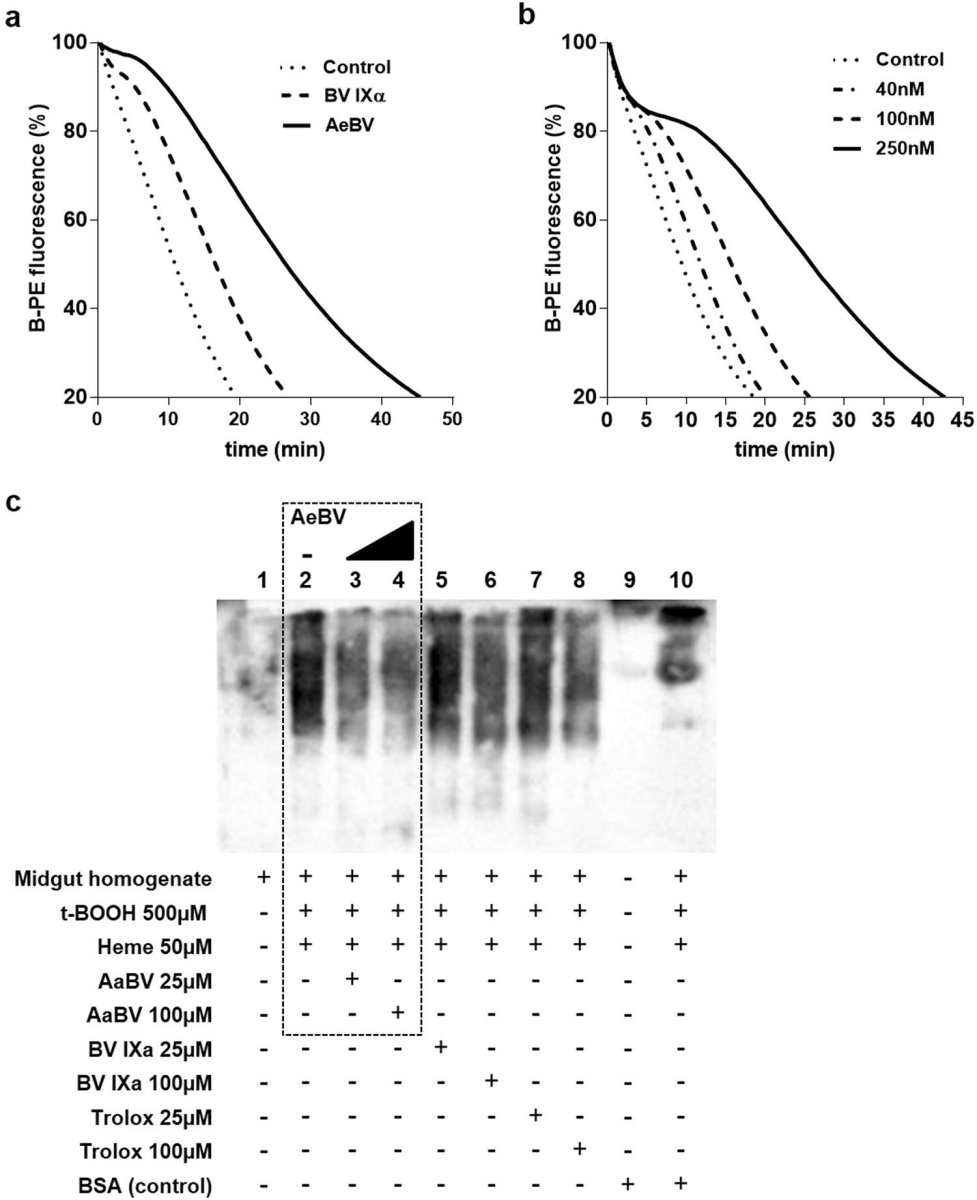
Antioxidant activity of AeBV. *B*-Phycoerythrin (B-PE) oxidation, in a prooxidant system, can be measured by the loss of its natural fluorescence in a fluorimeter. (**a**) Biliverdin IX-α and AeBV inhibit *B*-Phycoerythrin (B-PE) oxidation initiated by 4 mM ABAP, at the same concentration (250 nM). (**b**) Effect of AeBV is dose-dependent. (**C**) AaBV protects the midgut against protein oxidation *in vitro*. Midgut homogenates were incubated with heme and t-butyl hydroperoxide in the presence or absence of AaBV (evidenced in dashed square) – western-blot of *A*. *aegypti* midguts homogenate after incubations, using specific antibodies against protein carbonyl-DPNH products. Gel image was cropped and processed to improve clarity – original full-length blot/gel is presented in Supplementary Fig. S2.

It is well known that biliverdin (BV) and bilirubin (BR) are reducing molecules, acting as antioxidants *in vitro* and *in vivo*. Since in mammals BV is rapidly converted to BR by biliverdin reductase, most studies have been performed with BR. However, in *A*. *aegypti*, BV produced by cleavage of the heme porphyrin ring is further converted to a biglutaminyl-biliverdin IX alpha (AeBV)^9^. Similarly to BV IXα, AeBV showed antioxidant activity, as demonstrated by the delay of fluorescence decay caused by B-PE oxidation induced by the addition of the pro-oxidant ABAP. (Fig. 3a). The antioxidant activity of AeBV was also dose-dependent (Fig. 3b).

Considering that the midgut epithelium is potentially subjected to an oxidative challenge imposed by blood digestion and the release of high amounts of heme in the lumen, the antioxidant capacity of AeBV could be vital to protect midgut proteins from oxidation. In fact, as shown in Fig. 3c, protein oxidation was very extensive in the midgut extracts incubated in the presence of t-BOOH and heme, when compared to non-incubated samples. However, it could be partially reduced by the presence of AeBV at both tested concentrations. Similar reductions were observed with the samples treated in the presence of BV IXα and TROLOX, two well-known antioxidants. These results suggest that AeBV may represent a physiological antioxidant system, protecting midguts against protein oxidation generated by heme.

### Non-canonical transcriptional control of AeHO

**Figure 4:**
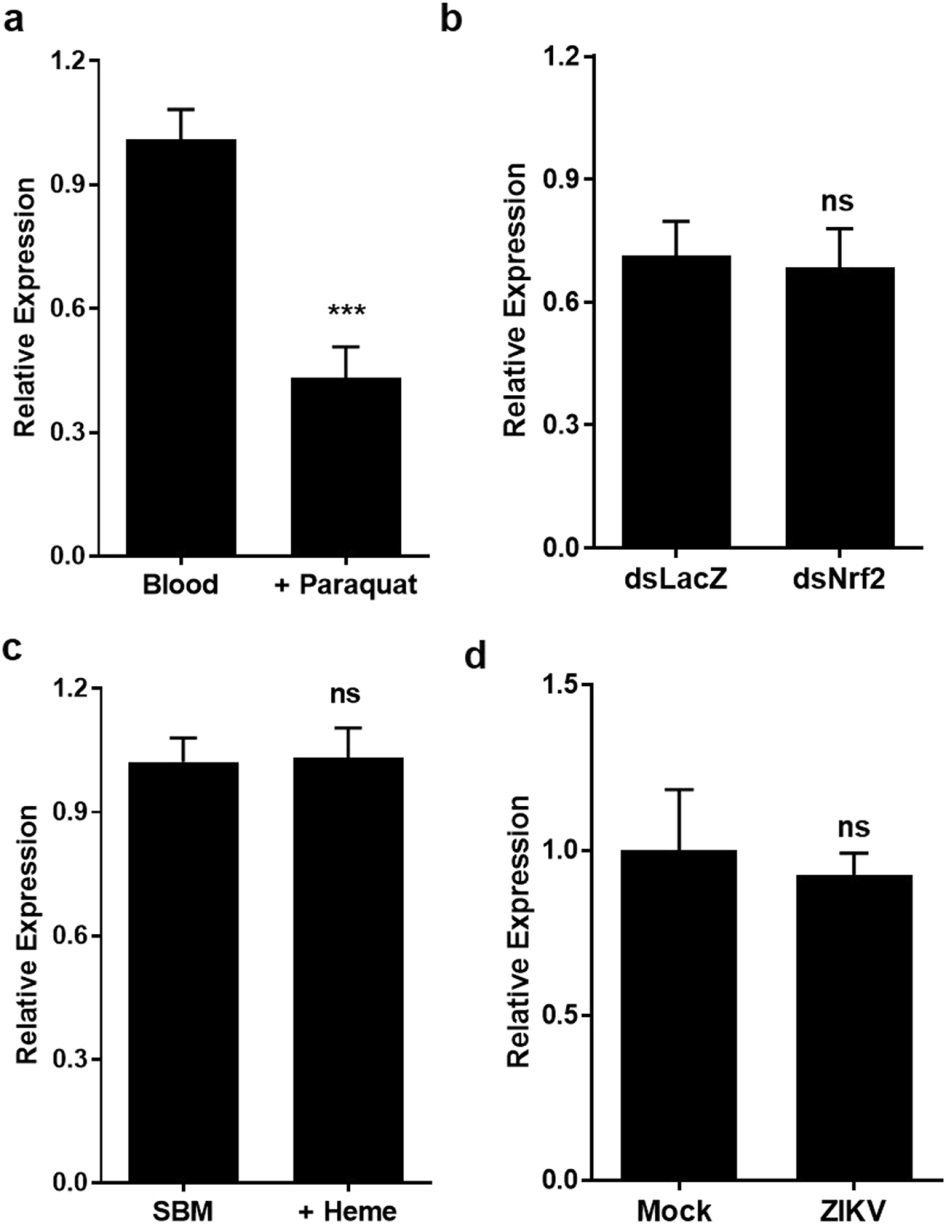
AeHO expression is not increased upon oxidative and viral challenges. Midgut expression of HO by real-time qPCR in (**a**) mosquitoes fed with blood supplemented or not with 500 µM paraquat; (**b**) paraquat-fed (1 mM) dsNrf2-injected mosquitoes; (**c**) mosquitoes fed with substitute blood meal (SBM) with or without 50 μM heme; (**d**) mosquitoes infected with ZIKV (compared to mock control). Results are pools of at least 3 independent experiments. Error bars indicate the standard error of the mean. Statistical analyses were performed by Student’s t-test.

HO is one of the major components of the response against several types of cellular stress. As a rule, mammalian HO-1 is induced by various stimuli such as oxidative stress and inflammation^16^. Surprisingly, in mosquito midguts, addition of paraquat, a compound that results in increased intracellular formation of superoxide anion^17^, leads to a decrease in AeHO gene expression (Fig. 4a). In addition, depletion through RNAi of the Nrf2, a master eukaryotic redox-active transcriptional regulator^18^ that has been extensively related to the induction of HO-1 expression^19^ (Fig. S1), has no effect on the AeHO mRNA levels (Fig. 4b).

Since AeHO transcript levels increase following a blood meal, we sought to verify if heme itself would account for this upregulation. To test this, we fed the insects with a protein-rich chemically defined artificial diet (Substitute Blood Meal, SBM), supplemented or not with heme^20^. Also unexpectedly, heme was not able to induce AeHO expression at these conditions (Fig. 4c).

In mammalian cells, induction of HO-1 has been recently recognized as a mediator of cellular protection against Zika virus (ZIKV) infection^21^. However, in the midgut of ZIKV-infected mosquitoes, AeHO gene expression remains unaltered (Fig. 4d).

**Figure 5:**
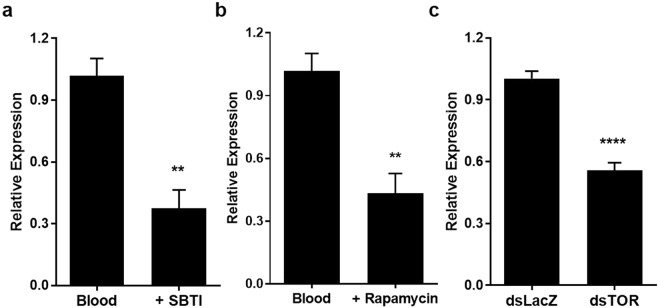
Amino acid-induced TOR regulatory cascade increases AeHO levels upon blood digestion. Midgut expression of HO by real-time qPCR in (**a**) mosquitoes fed with blood supplemented or not with 100 µM SBTI; (**b**) mosquitoes fed with blood supplemented or not with 20 µM rapamycin, and (**c**) blood-fed dsTOR-injected mosquitoes. Results are pools of at least 3 independent experiments. Error bars indicate the standard error of the mean. Statistical analyses were made by Student’s t-test.

Since AeHO in not canonically regulated, yet it is still induced throughout blood digestion, we investigated whether a nutrient sensor – such as the kinase Target of Rapamycin (TOR) – mechanism would be involved. In fact, the blood-meal increase in AeHO expression is partially blocked when protein digestion is impaired by ingestion of SBTI, a trypsin inhibitor (Fig. 5a). We further tested whether the amino acid-sensing target of rapamycin (TOR) pathway is involved in the control of AeHO transcription. In fact, inhibition of TOR, either by rapamycin (Fig. 5b) or by RNAi specific TOR depletion (Figs 5c and S1) was able to decrease blood-meal induced AeHO expression. These findings indicate that amino acid stimulation of the TOR regulatory cascade increases AeHO levels in the mosquito midgut upon blood digestion.

## Discussion

HO belongs to a large family of stress proteins whose transcriptional regulation also responds to varied types of adverse environmental conditions^16^. HO catalyzes the first and rate-limiting step in the oxidative degradation of heme (Fe-protoporphyrin-IX) to produce carbon monoxide, free iron, and biliverdin-IXα^22^. Large amounts of heme are produced in the gut of hematophagous insects, due to proteolysis of dietary hemoglobin. Given that proper heme degradation is essential to avoid pro-oxidizing environments in cells, it is expected that HO may be relevant in the adaptation of blood-feeding organisms to their heme-rich diet. Upon binding to the HO apoprotein, the heme molecule serves as the substrate and catalytic cofactor in its own degradation, and is generally considered as the prototypical inducer of HO expression^23^. Here we show that the transcriptional profile of AeHO matches the appearance of biliverdin – that has antioxidant properties – in the mosquito midgut. However, AeHO appears to be under an unexpected mode of transcriptional control, being regulated by a nutrient-sensing pathway instead of the canonical stress-response pathway.

While three isoforms of HO have been identified in mammals^8^, only a single gene coding for a HO is found in the *Aedes aegypti* genome. The identified gene AAEL008136, here named *Aedes aegypti* heme oxygenase (AeHO), encodes a polypeptide with basic residues that are considered essential to allow enzymatic cleavage of the porphyrin ring (Fig. 1). There is a high degree of evolutionary conservation of the heme-degrading enzymes with HO homologues identified in bacteria, fungi, and plants^24–26^. Generally, the mechanism of HO-catalyzed heme cleavage into an α-isoform of biliverdin, CO and iron is conserved between mammals and these organisms^19^. Among insects, however, there are some notable differences. The *D*. *melanogaster* HO-1 homolog (dHO), in contrast to mammalian HOs, is not alpha-specific, producing also the IX beta and IX delta isomers of biliverdin. Furthermore, the reaction rate is slower than that of mammalian HOs^12^. In blood-feeding insects, the tetrapyrole end products of heme degradation have acquired unique features important to facilitate their elimination. After a blood meal, in the gut of *R*. *prolixus* and *A*. *aegypti*, biliverdins conjugated to specific amino acids, cysteine and glutamine, respectively, are generated^9,10^. These further modified structures increase the solubility of biliverdin, converting it to a molecule easier to excrete. This suggests that heme degradation in blood-feeding insects evolved to be specialized in the production of massive amounts of biliverdin, generated upon blood digestion. We have demonstrated that heme degradation produces high amount of AeBV molecules that show significant antioxidant activities *in vitro* (Fig. 3). Considering that AeBV is produced and excreted in the midgut lumen during the course of digestion (Fig. 2e), we can suggest that heme degradation by HO represents a primary defense mechanism against heme toxicity: first, by reduction of the prooxidant heme levels and second by the production of a hydrosoluble antioxidant compound that may protect the gut epithelium against the oxidative challenge triggered by blood digestion.

HO is expressed even in stages and tissues that do not face high levels of heme in the diet or environment (Fig. 2a,b), suggesting that it is required to basal heme metabolism of that stage/tissue, probably been involved in the controlled of the intracellular free iron levels or in the production of CO that can act as a second messenger^16^. A similar result was obtained by a previous group, revealing that HO expression is required to the normal development of the non-hematophagous fruit fly *D*. *melanogaster*^27^.

Maximal expression of AeHO in the midgut occurs from 24 h to 42 h after the blood meal (Fig. 2c), when most of the ingested protein has already been degraded^28^. Monitoring the production of AeBV showed that this end product of the heme degradation pathway increased in the gut almost linearly until 24 h, when it starts to decline, which can be explained by its appearance in the feces (Fig. 2e,f). The total amount of AeBV (in the midgut content and in the feces) remains relatively constant after 24 h post blood meal, suggesting that the period of most intense enzymatic activity of the AeHO is, in fact, occurring simultaneously with the release of heme from dietary hemoglobin. The peritrophic matrix (PM) is a protective barrier with heme-binding activity, secreted by the midgut epithelium, composed by chitin, acidic polysaccharides and proteins, which are synthesized *de novo* in response to blood feeding^29,30^. During the initial hours of blood digestion, it is possible that the growing PM is not able to keep pace with the rate of hemoglobin hydrolysis due to elevated trypsin activity. Therefore, if the PM has not yet fully developed its capacity to isolate heme during the initial steps of digestion, a significant amount of heme would reach the midgut epithelium and AeHO activity would be responsible for counteracting heme toxicity. Unfortunately, all attempts to produce AeHO gene silencing by RNAi were unsuccessful (not shown).

Recent work has confirmed the existence of an enzyme with HO activity in *An*. *gambiae*, and its functional inhibition in adult females (by means of chemical inhibitors) has led to a dose dependent decrease in oviposition^13^. This correlates well with the data presented here, which show an ovary-specific peak of HO expression at the termination of oogenesis (Fig. 2d). Together with our recent report that silencing of HO shows a deleterious effect on oviposition and egg viability in the hematophagous insect *R*. *prolixus*^31^, we hypothesize that the protective role for HO in blood-feeding insects goes beyond digestion, being crucial for their adaptation to anautogeny as a reproductive strategy.

It is well known that the expression of mammalian HO-1 is induced by its substrate, heme, but its expression can also be strongly up-regulated in response to various stimuli related to cellular stress and pro-oxidant signals (reviewed in^32^). In the mosquito midgut, transcription of the HO gene is regulated both by ingestion of the meal and by formation of digestion products, as trypsin inhibition decreases HO transcript levels (Fig. 5a). However, unexpectedly, the ingested heme is not the trigger in this regulatory cascade (Fig. 4c). While this unresponsiveness of HO to heme has been briefly described in cultured *Drosophila* cells before^27^, here we consolidate this feature as part of an intricate physiological regulation of the blood digestion process that couples spatiotemporal hemoglobin catabolism, heme degradation and PM formation in the mosquito midgut.

Another unique feature of AeHO is that, in contrast to mammalian HO-1 which is over expressed upon ROS stimuli, AeHO showed an opposite profile when the blood meal was supplemented with a ROS generator molecule, paraquat (Fig. 4a). Nrf2, a basic leucine zipper transcription factor, is a master regulator of the transcriptional response to oxidative stress that, upon activation, regulates the expression of genes coding for anti-oxidant, anti-inflammatory and detoxifying proteins. For most organisms it is generally accepted that HO is one of the key Nfr2 pathway-regulated genes (reviewed in^18^). In *A*. *aegypti*, silencing of the Nrf2-homolog did not impair AeHO transcription (Fig. 4b) but significantly altered other stress-related genes^33^. Aside from a possible post-transcriptional regulation of this enzyme, as observed for mammals^34^, these results support the hypothesis of multi-transcriptional control of AeHO expression.

The mosquito heme degradation system seems to follow some canonical aspects in its operation, but also involves a unique expression control, that seems related to the pressure imposed by a blood feeding habit. A blood meal provides a surplus of heme and iron (an important micronutrient for mosquitoes), coupled with a wide range of changes in the expression of genes related to iron/heme homeostasis. Ferritin (an iron-storage protein) is transcriptionally induced after a blood meal, contributes to sequester iron released from heme digestion, and accumulates into the eggs, preventing oxidative damage during embryo development (reviewed in^35^). Therefore, heme degradation is supposed to be protective only when coupled to efficient iron sequestration by ferritin, in order to avoid production of reactive oxygen species by Fenton reaction^5^. In fact, in the mosquito, genes encoding ferritin and catalase, that counteract the production of hydroxyl radicals, respectively by isolating iron and scavenging hydrogen peroxide, are differentially regulated in the midgut up to 24 hours after a blood meal^36^ and are proposed to be part of a heme and paraquat-induced response^37^. Accordingly, a negative feedback loop seems to be operating in the mosquito - in the presence of high levels of ROS, the midgut cells can decrease heme degradation, by inhibiting HO expression. This might offer some adaptive value, by avoiding increase in the intracellular levels of free iron formed during HO catalysis, which can take part in deleterious reactions. Otherwise, with an impaired redox balance, cleavage of the heme ring by HO would represent a pro-oxidant event for the insect.

The highly conserved target of rapamycin (TOR) pathway plays a critical role in regulation of translation by conveying extracellular nutritional conditions as an amino acid-sensing system^38,39^. TOR signaling is a key pathway linking blood digestion and egg development in *A*. *aegypti*^40,41^. This regulation is finely tuned to the nutritional requirements of the mosquito, and occurs at transcriptional and post-translational levels^42,43^. Since blood digestion impairment (Fig. 5a) is able to suppress AeHO gene expression, we tested whether this nutrient-sensing pathway could act upstream in the transcriptional regulation of AeHO. In fact, the use of rapamycin together with the blood meal, and the specific TOR depletion by RNAi (Fig. S1) were both effective in down regulating AeHO after feeding (Fig. 5b,c). This amino acid-dependent nutrient signaling has been associated with regulation of transcripts linked to mosquito vitellogenesis and digestion, in the fat body and the midgut, respectively^40,41^. The TOR-mediated cascade includes a GATA factor, which is the specific transcriptional activator of the vitellogenin gene. GATA binds specifically to GATA-binding sites in the proximal promoter region of the vitellogenin gene to activate its expression^44^. Indeed, we also found putative GATA-binding motifs in the promoter region of the AeHO gene (Fig. S1), which could indicate that this factor is the final downstream step in the TOR-mediated regulation of AeHO. Despite the fact that HO-1 in mammals is induced by rapamycin in injury models^45–47^, we consider that in such examples this enzyme mediates the protective effects of rapamycin, exerting its expected cytoprotective role. However, TOR signaling in hematophagous insects has a fundamental role in supporting metabolic adaptations important for blood feeding. For instance, in *R*. *prolixus*, blood meal amino acids decrease ROS levels in the midgut immediately after feeding, through the TOR pathway^48^. In *A*. *aegypti*, this ROS minimizing event is mediated by heme itself^49^, which strongly indicates that coupling the transcriptional regulation of AeHO with protein digestion can be an additional element in the intricate heme-driven response that occurs in the mosquito midgut upon blood feeding.

In mammals HO-1 expression and activity have been associated with anti-inflammatory, antioxidant, anti-apoptotic, and anti-proliferative effects that underlie tissue-protective responses under pro-inflammatory conditions (reviewed in^32^). Additionally, during viral infections, HO-1 has been reported to display noteworthy antiviral activity against a wide variety of viruses, including Zika and dengue viruses (Huang *et al*., 2017 and reviewed in^50^). Despite the fact that ZIKV infection was not capable of altering AeHO gene expression (Fig. 4d), further genetic knockdown studies are required to directly investigate the role of AeHO in the context of mosquito infection and its influence on the redox homeostasis and vectorial adaptation of *A*. *aegypti*.

**Figure 6:**
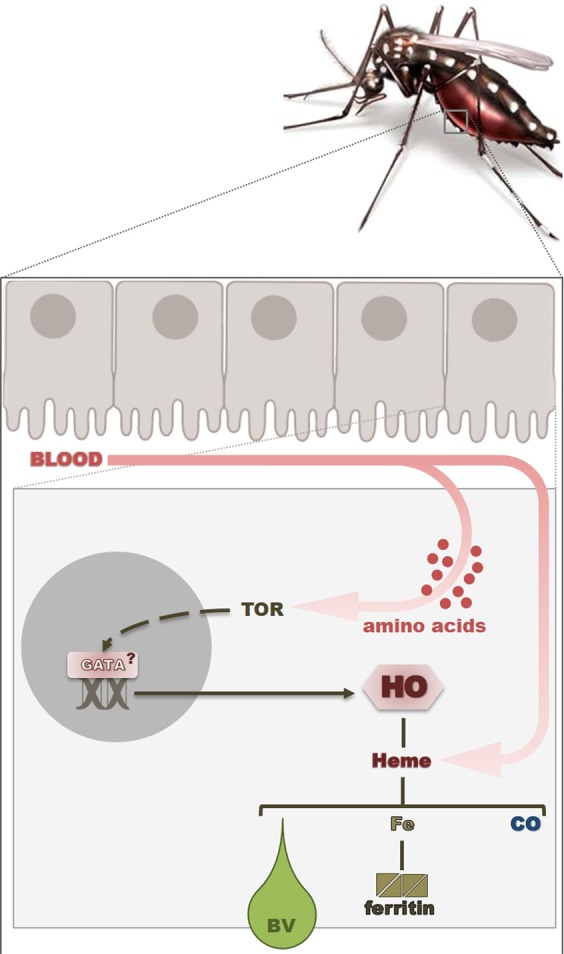
Overview of the non-canonical and coupled with digestion transcriptional regulation of heme oxygenase in *Aedes aegypti* midgut. Schematic representation of a female of *A*. *aegypti* mosquito after a blood meal and in detail, the inside of a midgut cell – amino acids activate target-of-rapamycin (TOR) pathway, which induces the transcription of AeHO (presumably through the GATA factor) resulting in heme degradation and production of AeBV, thus contributing to gut homeostasis and hematophagic adaptation.

Taken together, our results suggest a fundamental and novel role for HO in the mosquito. It is well established that heme degradation has an antioxidant role, not only by removal of the pro-oxidant free heme but also due to the antioxidant properties of biliverdin. Furthermore, we show that in the *A*. *aegypti* midgut, the heme degradation after a blood meal is fine-tuned through a nutrient-sensing regulation, being as essential as the blood digestion itself. The enzymatic heme degradation by AeHO seems to play a key role in the adaptation of *A*. *aegypti* to blood feeding, and its unusual transcriptional regulation may act as an evolutionary response to compensate for the massive ingestion of heme (Fig. 6).

## Methods

### Ethics statement

All animal care and experimental protocols were conducted in accordance with the guidelines of the Committee for Evaluation of Animal Use for Research (Federal University of Rio de Janeiro, CAUAP-UFRJ) and the National Institutes of Health (NIH) Guide for the Care and Use of Laboratory Animals (ISBN 0-309-05377-3). Dedicated technicians in the animal facility at the Instituto de Bioquímica Médica Leopoldo de Meis (IBQM) carried out all protocols related to rabbit husbandry under strict guidelines to ensure careful and consistent animal handling. The protocols were approved by CAUAP-UFRJ under registry #IBQM155/13.

### HO sequence analyses

Multiple sequence alignment was constructed using the Clustal Omega web tool (http://www.ebi.ac.uk/Tools/msa/clustalo/). The evolutionary history was inferred using the Neighbor-Joining method^51^ and the analyses were conducted in MEGA7^52^.

### Mosquitoes

*Aedes aegypti* (Red Eye strain) were raised in a mosquito rearing facility at the Federal University of Rio de Janeiro, Brazil, under a 12 h light/dark cycle at 28 °C and 70–80% relative humidity. Larvae were fed with dog chow^20^, and adults were maintained in a cage and given a solution of 10% sucrose *ad libitum*. Four to seven day-old females were used in the experiments.

### Mosquito meals

Female mosquitoes were artificially fed with the following different diets: (1) heparinized-rabbit blood with or without 500 µM paraquat or 20 µM rapamycin; (2) Substitute Blood Meal (SBM)^20^ with or without 50 µM heme. Feeding was performed using water-jacketed artificial feeders maintained at 37 °C sealed with parafilm membranes. For RNA sample preparation, midguts (15–20) were dissected 24 h after feeding.

### Mosquito gene knock-down by RNAi

Double-stranded RNA (dsRNA) was synthesized from fragments amplified from cDNA of whole mosquitoes using specific primers containing a T7 tail. The *in vitro* dsRNA transcription reaction was adapted from a tRNA transcription protocol^53^. Briefly, it was performed at 37 °C for 12 h in a buffer containing 40 mM Tris•HCl (pH 8.0), 22 mM MgCl2, 5 mM DTT, 2 mM spermidine, 0.05% BSA, 15 mM guanosine monophosphate, 7.5 mM of each nucleoside triphosphate, PCR amplified template DNA (0.1 μg/μL) and 5 µM of a previously purified T7 RNA polymerase. The transcribed dsRNA was treated with DNAse at 37 °C for 30 minutes and precipitated using 1:10 (v/v) 3 M Sodium acetate pH 5.2 and 1 (v/v) of isopropanol. The pellet was washed twice with 70% ethanol and then eluted in water to reach a final concentration of 3 µg/µL. Mosquitoes were injected in the thorax with the double-stranded RNA (0.4 µg) and were blood-fed 48 h post injections. LacZ gene was used as a non-related dsRNA control and was amplified from a plasmid containing a cloned LacZ fragment. The oligonucleotides sequences used in dsRNA synthesis of Nrf2 and TOR can be found elsewhere^33,40^.

### Viral infections

Infection procedures were performed as described previously (Bottino-Rojas *et al*., 2018). Briefly, Zika viral stocks (ZIKV-BR_PE_^54^) were propagated in C6/36 cells maintained in Leibovitz-15 medium supplemented with 5% fetal bovine serum, 1% nonessential amino acids, 1% penicillin/streptomycin and tryptose (2.9 g/liter). Females were infected in an artificial blood meal containing a 1:1 mix of rabbit red blood cells and L-15 medium containing Zika virus. Midguts were dissected at 5 days post-blood meal and subjected to RNA isolation.

### RNA isolation, conventional and quantitative PCR analysis

Total RNA was isolated from 20–30 insects at different developmental stages (first and third instars larvae, and pupae), whole bodies of adult males and females and from midgut epithelium, ovary, head, Malpighian tubules, thorax and abdomen (carcass) of blood fed females using TRIzol (Thermo Fisher Scientific, Waltham, MA USA) according to the manufacturer’s protocol. Complementary DNA was synthesized using the High-Capacity cDNA Reverse transcription kit (Thermo Fisher Scientific, Waltham, MA USA). PCR was performed using Taq DNA polymerase (Thermo Fisher Scientific, Waltham, MA USA) for 35 cycles (30 seconds at 94 °C, 30 seconds at 60 °C and 30 seconds at 72 °C) in a thermocycler GeneAmp PCR System 2400 (Thermo Fisher Scientific, Waltham, MA USA). Both fragments obtained were about 100 bp in length. PCR products were separated on a 2% agarose ethidium bromide stained gel.

The Real time qPCRs were performed with the StepOnePlus Real Time PCR System (Thermo Fisher Scientific, Waltham, MA USA) using the Power SYBR-green PCR Master Mix (Thermo Fisher Scientific, Waltham, MA USA). The Comparative Ct Method (Livak and Schmittgen, 2001) was used to compare the changes in the gene expression levels. The *A*. *aegypti* ribosomal protein 49 gene (RP49) was used as reference gene, based on previous data^55^. All oligonucleotides sequences used in qPCR assays are available in the Supplementary Material.

### Pigment extraction

For AeBV pigment identification and purification, females were fed with rabbit blood. Fifty midguts were dissected under 50% ethanol, transferred to 10 mM sodium phosphate, 0.15 M NaCl, pH 7.4 (PBS), homogenized and centrifuged for 15 minutes at 12,000 × g. Supernatants were dried under vacuum, kept protected from light, and stored at −20 °C until analysis and purification by HPLC.

### HPLC fractionation

HPLC was performed on a Shimadzu CLC-ODS C18 column (15 mm × 22 cm) using a Shimadzu LC-10AT device (Tokyo, Japan), equipped with a diode array detector (SPD-M10A). Chromatography analysis was performed using 5% acetonitrile with 0.05% trifluoroacetic acid (TFA) as solvent, at a flow rate of 0.4 mL/minutes. Before injection, dried samples were diluted in 10% acetonitrile with 0.05% TFA and centrifuged for 15 minutes at 12,000 × g. Ten minutes after sample injection a 40 minutes linear acetonitrile gradient (5–80%) was applied, followed by 20 minutes of 80% acetonitrile. Supernatants were dried under vacuum, and stored at −20 °C protected from light until use as described above. AeBV peak area data were taken for statistical analyses.

### B-phycoerythrin oxidation protection assay

Loss of B-phycoerythrin (B-PE) fluorescence as a result of oxidation by peroxyl radicals generated by thermal decomposition of ABAP (2,2′-azobis (2-amidinopropane) dihydrochloride) was performed with a protocol modified from^56^. B-PE (Sigma) stocks were prepared in 0.01 M NH_4_HCO_3_ pH 5.0 to a final concentration of 2.08 × 10^−6^ M and stored at 4 °C under light protection. Stock solutions of the pro-oxidant ABAP (40 mM), and of the water soluble antioxidants ascorbic acid, TROLOX (Sigma), and AeBV (1 mM) were freshly prepared in 0.075 M NaH_2_PO_4_/KH_2_PO_4_ buffer pH 7.0. BV IXα solution (1 mM) was prepared in DMSO (Sigma). For B-PE oxidation assays with BV IXα, AeBV was also dissolved in DMSO. The B-PE oxidation reaction was started by addition of 10 µL of ice-cold ABAP stock solution to a cuvette containing the reaction mixture (1 mL) composed of B-PE (1,65 × 10^−8^ M) and the antioxidants (250 nM) in 0.075 M NaH_2_PO_4_/KH_2_PO_4_ buffer pH 7.0, at 37 °C. Control samples were prepared in the absence of antioxidants. DMSO was added to the controls of BV IXα assays. Fluorescence was monitored in a VARIAN Cary Eclipse Fluorescence Spectrophotometer (Agilent Technologies, CA, USA) Fluorimeter for 60 minutes, excitation was measured at 540 nm and emission at 565 nm. For data analyses, fluorescence emission values were expressed as percentage of the initial fluorescence of B-PE.

### Detection of protein carbonylation by western blot

Heme stock solutions (5 mM) were prepared as following: 3.27 mg of hemin was dissolved in 200 µL of 0.1 N NaOH and vigorously vortexed for 10 minutes. 800 µL of PBS pH7.4 was added followed by vigorous vortexing for 5 minutes. The solutions of 10 mM tert-butylhydroperoxide (t-BOOH) (Sigma) and TROLOX were prepared in Milli-Q water. Biliverdin IX alpha (BV IXα) stocks were prepared in 0.1 M of NaOH. The AeBV stock was prepared as described above. All the solutions were freshly prepared.

A single homogenate (female midguts extract) from 40–50 midguts was prepared as described in the item pigment extraction. We challenged midgut extract samples with a reaction mixture, composed by t-BOOH in the presence of heme, a system that is able to produce alkyl and peroxyl radicals. Carbonyl groups derived from oxidation of midgut proteins were subjected to derivatization with DPNH (2,4-dinitrophenylhydrazine) and detected by western-blot using anti-DPNH antibodies.

Samples of the guts homogenate (30 µg of total protein content) were incubated in PBS, pH 7.4 with 500 µM t-BOOH and 100 µM heme, in the absence or presence of AeBV (25 or 100 µM), for 60 minutes at 37 °C. Samples from the same homogenate were also incubated with 500 µM t-BOOH and 100 µM heme, in the absence or presence of Biliverdin IXa (25 or 100 µM), and the antioxidant TROLOX as controls. The oxidation reactions were stopped by addition of 50 mg/mL of the antioxidant butyl-hydroxytoluene (BHT) and SDS-PAGE loading buffer. BSA incubated with heme and t-BOOH or non-incubated midgut homogenates and BSA samples were used as positive and negative controls, respectively. Samples were subjected to 15% SDS-PAGE and transferred to PVDF membranes. Proteins were derivatized by treatment with 0.1 mg/mL DNPH in 2 N HCL for 30 minutes at RT. After incubation, membranes were washed 3 times with 2 N HCL and 5 times with 100% Methanol. Finally, membranes were rinsed with PBS and incubated with rabbit anti-DPNH antibody (Thermo Fisher Scientific, Waltham, MA USA) diluted 1:5000. Sequentially, anti-rabbit IgG conjugated to horseradish peroxidase and ECL Western blotting detection system was used (Thermo Fisher Scientific, Waltham, MA USA) according to the manufacturer’s instructions. This analysis was followed by visualization on X-ray film (Amersham Hyperfilm ECL, Buckinghamshire, United Kingdom). Membranes were kept under light protection during all procedures described above (modified from Wehr and Levine, 2012). BSA (about 2 µg) was used as positive control for the oxidation system. BV IXα and TROLOX (25 or 100 µM) (Sigma) were used as standard antioxidants for this antioxidant capacity assay.

### Statistical analysis

All analyses were performed with GraphPad Prism Statistical Soſtware Package (Prism 6.0, GraphPad Soſtware, Inc., San Diego, CA). Asterisks indicate significant differences (***p < 0.001; **p < 0.01; *p < 0.05; ns = non-significant) and each used post-test analysis is described in its respective figure legend.

## Supplementary information

Supplementary informations

## Data Availability

All data generated or analyzed during this study are included in this published article (and its Supplementary Information Files).
