# Supplementary material for: Non-canonical transcriptional regulation of heme oxygenase in Aedes aegypti
Source: Sci Rep. 2019 Sep 24;9:13726. doi: 10.1038/s41598-019-49396-3 (PMC6760526; doi:10.1038/s41598-019-49396-3)
Supplement: Supplementary file 1 — Supplementary informations [file 41598_2019_49396_MOESM1_ESM.doc]

**Supplementary Information**

**Non-canonical transcriptional regulation of heme oxygenase in *Aedes aegypti***

**Vanessa Bottino-Rojas^1†^, Luiza O.R. Pereira^2†^, Gabriela Silva^1^, Octavio A. C. Talyuli^1^, Boris C. Dunkov^3^, Pedro L. Oliveira^1^ and Gabriela O. Paiva-Silva^1^***

^1^ Instituto de Bioquímica Médica Leopoldo de Meis, Universidade Federal do Rio de Janeiro, Rio de Janeiro, 21941-902, Brazil

^2^ Laboratório de Pesquisas em Leishmaniose, Instituto Oswaldo Cruz, FIOCRUZ, Rio de Janeiro, 21040-360, Brazil

^3^ Center for Insect Science, The University of Arizona, Tucson, AZ, 85721-0106, USA

* Corresponding author: *gosilva@bioqmed.ufrj.br*

^†^ These authors contributed equally to this work.

**Supplementary figures**


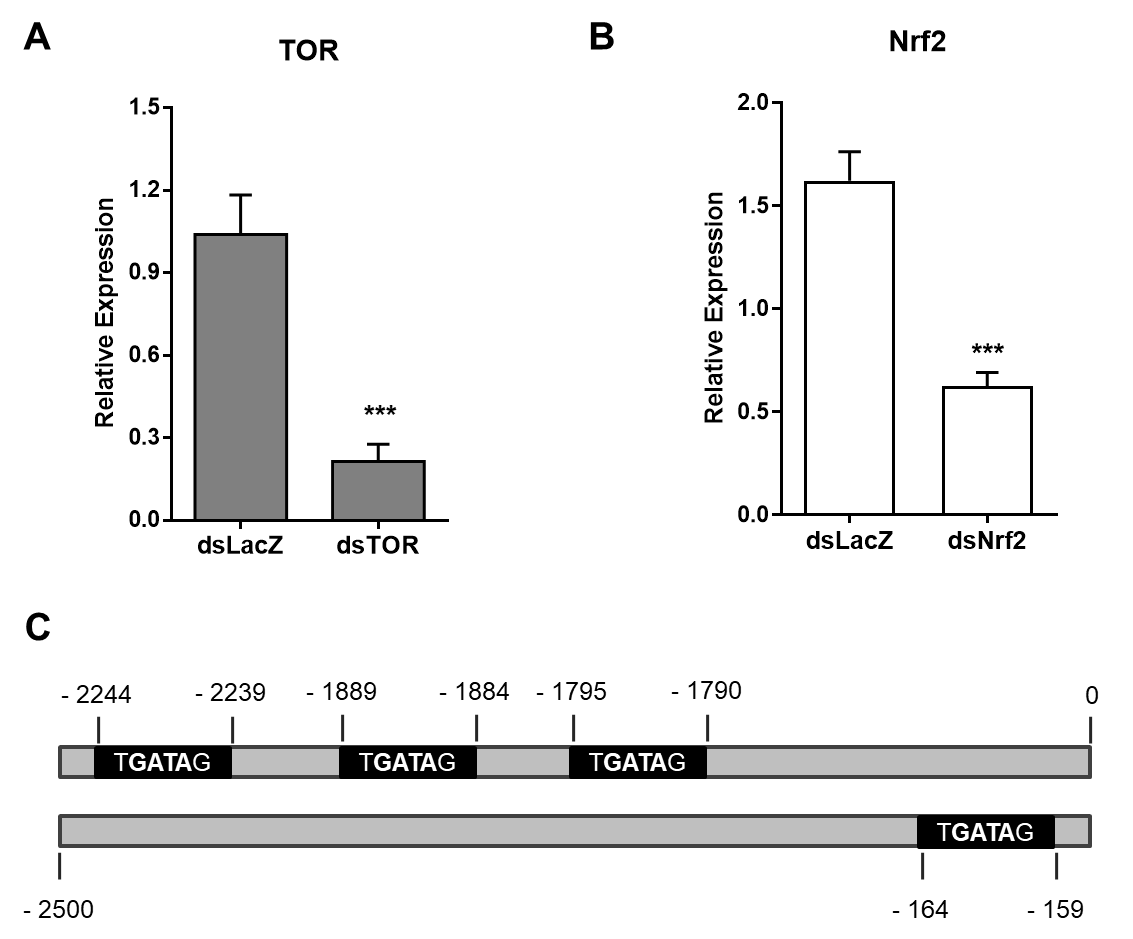


**Figure S1**

**(A)** dsRNA silencing efficiency of TOR and **(B)** Nrf2. **(C)** Schematic illustration of the GATA-binding motifs in the promoter region of AeHO. Numbers refer to nucleotide positions relative to the transcription start site.

**
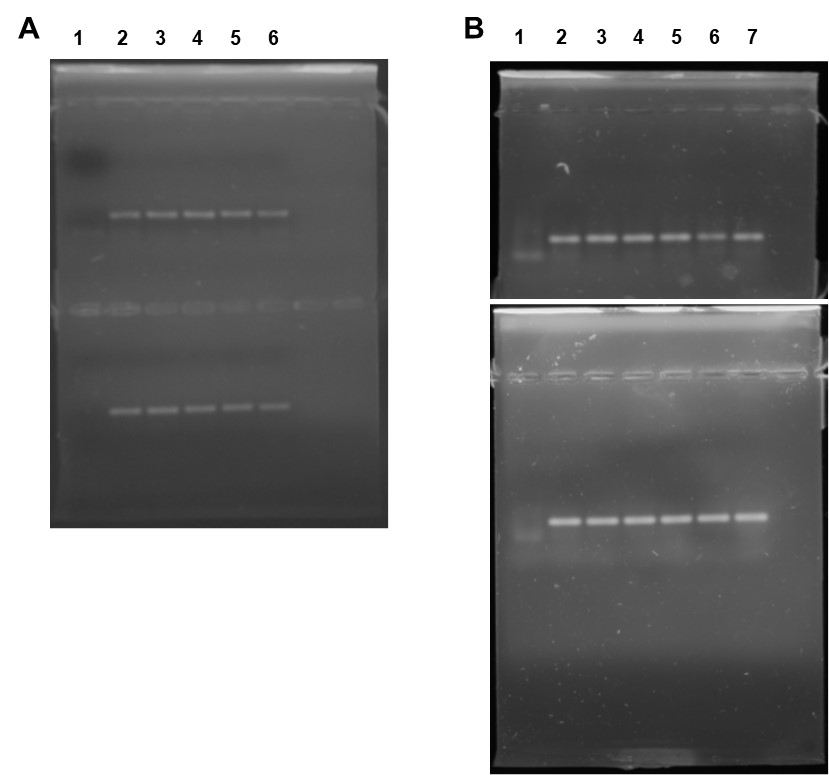
**

**Figure S2**

**(A)** Full-length gel (2% agarose ethidium bromide stained) presented in Figure 2A. Top: HO; Bottom: Rp49. Numbers read as follow: 1. CT (-) (negative control) / 2. Larvae (L1) / 3. Larvae (L4) / 4. Pupae / 5. Adult Female / 6. Adult Male. **(B)** Full-length gel (2% agarose ethidium bromide stained) presented in Figure 2B. Top: HO; Bottom: Rp49. Numbers read as follow: 1. CT (-) (negative control) / 2. Midgut / 3. Ovaries / 4. Malpighian tubules / 5. Head / 6. Thorax / 7. Abdomen (carcass).

**
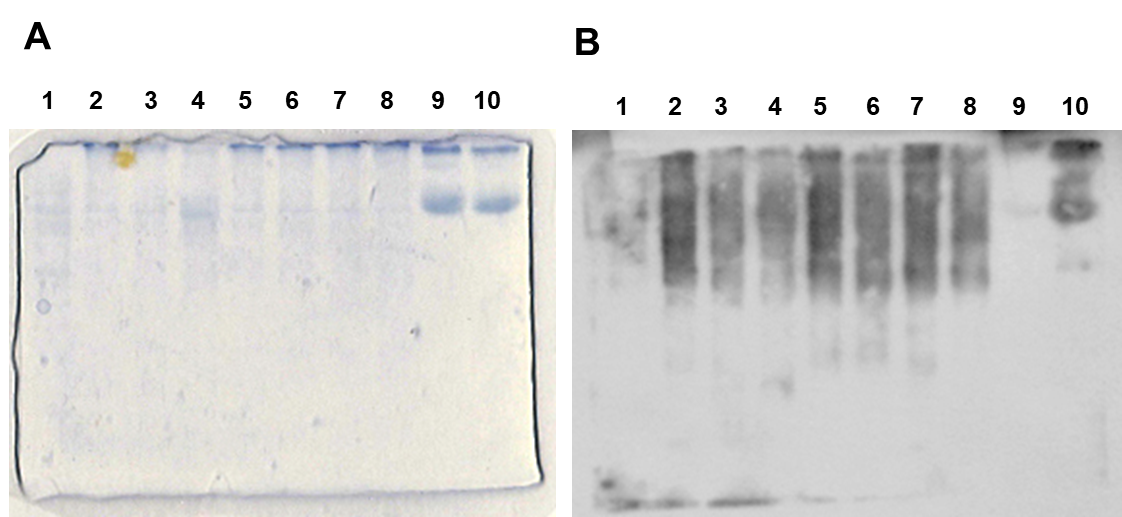
**

**Figure S3**

**(A)** Comassie-stained SDS/PAGE gel used for the western blot experiment presented as loading control; **(B)** Full-length blot presented in Figure 3C. Numbers read as follow: 1. Non-incubated midgut homogenate / 2. t-BOOH 500µM + heme 50µM / 3. t-BOOH 500µM + heme 50µM + AaBV 25µM / 4. t-BOOH 500µM + heme 50µM + AaBV 100µM / 5. t-BOOH 500µM + heme 50µM + BV IXα 25µM / 6. t-BOOH 500µM + heme 50µM + BV IXα 100µM / 7. t-BOOH 500µM + heme 50µM + Trolox 25µM / t-BOOH 500µM + heme 50µM + Trolox 100µM / 9. Non-incubated BSA (negative control) / 10. BSA + t-BOOH 500µM + heme 50µM (positive control).

**Supplementary materials and methods**

*Primers Designed for qRT-PCR Assays*

For qPCR amplification the following primers were used: HO forward 5'-CAAAACTTGCTTTCGCCC-3' and HO reverse 5'-GCAGGAAGTCATGCGATACA-3'. TOR forward: 5’-GCTGAAGAGCCCCTCGTC; TOR reverse, 5’-CACGTGCATGACGCTTTC; Nrf2 forward: 5’-CGCTACCGTCAGCTACCAAT; Nrf2 reverse: 5’-CGGCTCCCTCTAAGTGACTG. For RP49 (used as reference gene) primers were: forward 5'-ACAAGCTTGCCCCCAACT-3' and reverse 5'-CCGTAACCGATGTTTGGG-3'.

*Discovery of cis-regulatory elements*

The motif-based sequence analyses suite MEME^1^ was used to investigate the 2500 base-pairs (bp) adjacent to the 5’-end of the transcription start site of AeHO. FIMO tool^2^ was used to search for matches to GATA-binding motifs.

**References**

1. Bailey, T. L. *et al.* MEME Suite: Tools for motif discovery and searching. *Nucleic Acids Res.* **37,** 202–208 (2009).

2. Grant, C. E., Bailey, T. L. & Noble, W. S. FIMO: Scanning for occurrences of a given motif. *Bioinformatics* **27,** 1017–1018 (2011).
